# Functionalized Microstructured Optical Fibers: Materials, Methods, Applications

**DOI:** 10.3390/ma13040921

**Published:** 2020-02-19

**Authors:** Timur Ermatov, Julia S. Skibina, Valery V. Tuchin, Dmitry A. Gorin

**Affiliations:** 1 Skolkovo Institute of Science and Technology, 3 Nobelya str., 121205 Moscow, Russia; 2 SPE LLC Nanostructured Glass Technology, 101 50 Let Oktjabrja, 410033 Saratov, Russia; skibinajs@yandex.ru; 3 Research Educational Institute of Optics and Biophotonics, Saratov State University, 83 Astrakhanskaya str., 410012 Saratov, Russia; tuchinvv@mail.ru; 4 Interdisciplinary Laboratory of Biophotonics, Tomsk State University, 36 Lenin’s av., 634050 Tomsk, Russia; 5 Laboratory of Laser Diagnostics of Technical and Living Systems, Institute of Precision Mechanics and Control of the Russian Academy of Sciences, 24 Rabochaya str., 410028 Saratov, Russia

**Keywords:** microstructured optical fibers, photonic crystal fibers, holey fibers, hybrid fibers, modification, functionalization, sensing

## Abstract

Microstructured optical fiber-based sensors (MOF) have been widely developed finding numerous applications in various fields of photonics, biotechnology, and medicine. High sensitivity to the refractive index variation, arising from the strong interaction between a guided mode and an analyte in the test, makes MOF-based sensors ideal candidates for chemical and biochemical analysis of solutions with small volume and low concentration. Here, we review the modern techniques used for the modification of the fiber’s structure, which leads to an enhanced detection sensitivity, as well as the surface functionalization processes used for selective adsorption of target molecules. Novel functionalized MOF-based devices possessing these unique properties, emphasize the potential applications for fiber optics in the field of modern biophotonics, such as remote sensing, thermography, refractometric measurements of biological liquids, detection of cancer proteins, and concentration analysis. In this work, we discuss the approaches used for the functionalization of MOFs, with a focus on potential applications of the produced structures.

## 1. Introduction

Starting from the first realization of microstructured optical fibers (MOFs) over twenty years ago [1,2], they have found numerous applications in optics [3], optogenetics [4], life science [5], plasmonics [6,7], and related fields [8,9]. Among others, biomedicine and biochemistry with their increasing demand for fast, precise, and easy sensing techniques became major stimuli for the development of optical fiber-based sensors (OFSs). Compared to bulk laboratory instruments, optical fibers possess unique properties (compact size, high flexibility, and optical path control, easy to use and low cost, mechanical stability, and immunity to electromagnetic and radiofrequency interferences) [10,11] which have defined their widespread use in the chemical and biochemical analysis [12]. Another reason to use OFSs is their high sensitivity to refractive index (RI) changes that enable precise concentration measurements and the detection of bioactive molecules with both low and high molecular weight.

In this work, we review the various types of functionalization techniques of MOFs that have enabled the improvement of their performance and have created new perspectives for the use of OFSs. We extensively describe the methods for integrating host materials inside the fibers for the cases of plasmonic nanoparticles and metal nanowires. The novel technique of layer-by-layer (LBL) [13] deposition, previously used mainly for the deposition of thin films onto planar substrates and adapted for the functionalization of fiber surfaces, in order to make the attachment of target molecules or particles possible, or for the creation of areas sensitive to a specific medium, is also considered [14,15,16]. This review concentrates on the different methods used for the modifications of optical fibers and highlights the novel functionalities which go beyond the manipulation of transmitted light, revealing the innovative applications of these structures, rather than investigating the origins of the underlying physical phenomena.

## 2. Microstructured Optical Fiber-Based Optical Sensors

**Figure 1:**
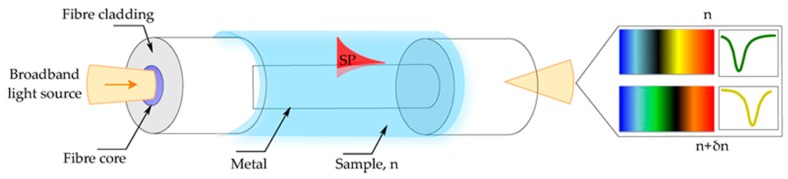
Optical fiber sensor based on surface plasmon resonance (SPR). Illustration of the working principle of the fiber-based SPR sensor. The shift of the plasmon resonant wavelength is associated with the change in the refractive index of the sample (δn) defining the resonant condition. Reproduced with permission from [21].

The recent developments in thin film deposition techniques have enabled the modification of fiber surfaces with nano-coating layers. This allows exploiting the phenomena of surface plasmon resonance (SPR) [7,17], localized surface plasmon resonance (LSPR) [18,19], and lossy mode resonance (LMR) [6] to detect changes of the surrounding medium by measuring the spectral shift of the plasmonic resonance [12,20,21]. However, the strong influence of uniformity and thickness of the metal coating on the sensing performance, together with complicated and high-cost thin-film deposition processes, inhibits optimal performance for these sensors [18,22]. Moreover, the necessity to cover the whole sensor surface hinders their efficient use with low analyte volumes, which is crucial for bio-applications (Figure 1).

**Figure 2:**
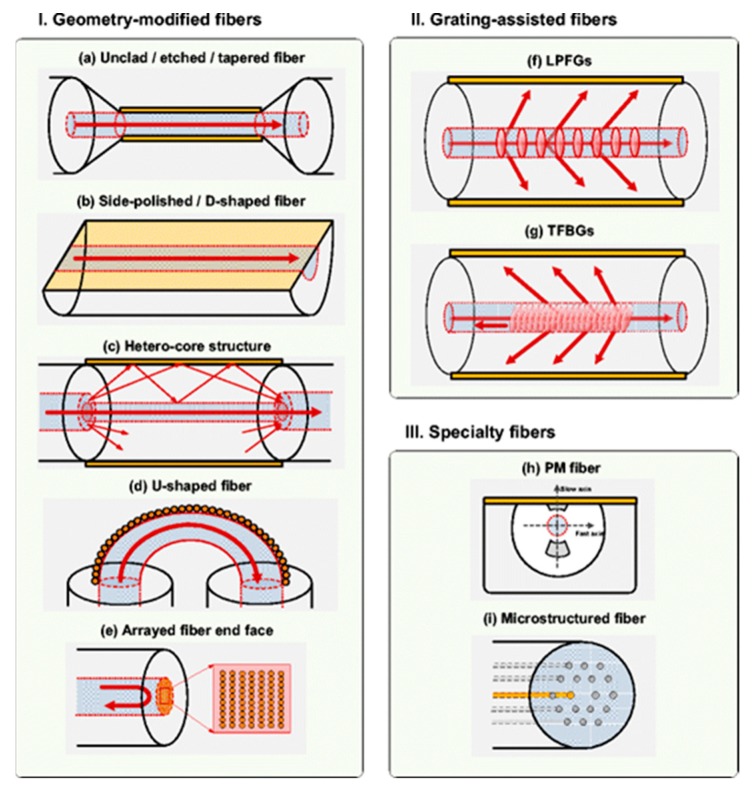
An overview of different structure-modified optical fibers. (**I**) Geometry-modified optical fibers: (**a**) Unclad, etched, (**b**) side-polished, D-shaped fibers, (**c**) hetero-core structures, (**d**) U-shaped fibers, and (**e**) arrayed fiber end face. (**II**) Grating-assisted fibers. (**III**) Specialty fibers. Reproduced with permission from [20].

Among the different structures of OFSs and related microstructures [23], sensors based on geometry-modified fibers (D-shaped, polished, etched, and tapered) [20,24,25,26], grating-assisted fibers [27,28,29], and MOF [30,31] represent three of the most widely developed OFS groups (Figure 2).

Nevertheless, the major challenge of the first two sensor structures is the low sensitivity for detecting small biomolecules and low analyte concentration. At the same time, surface modifications such as cladding removing, side polishing and fiber tapering, which lead to enhanced detection sensitivity, suffer from poor mechanical stability and complicated fabrication processes [22,32].

**Figure 3:**
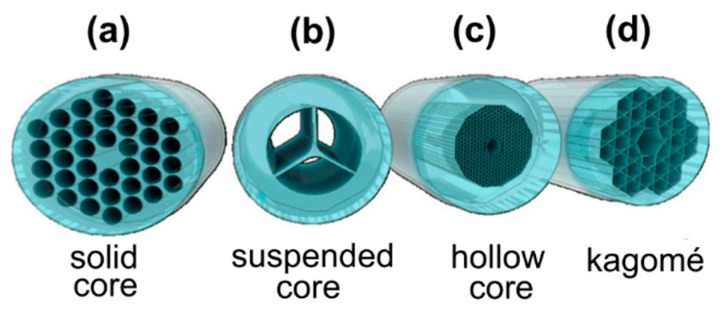
Illustration showing the different types of microstructured optical fiber-based sensors (MOFs): (**a**) Solid-core MOF, (**b**) suspended-core MOF, (**c**) hollow-core (HC)-MOF, (**d**) Kagomé HC-MOF. Reproduced with permission from [12].

Recently, MOFs or holey fibers, whose structure is defined by an array of air-channels, running through the whole fiber length, have been extensively studied, exploiting their unique characteristics for the creation of highly sensitive chemical and biological sensors with applications in biophotonics, chemistry, and life sciences. MOFs can either be divided into two main categories depending on their structure, hollow-core MOFs (HC-MOFs) and solid-core MOFs or distinguished depending on their light guidance principles (Figure 3).

In contrast to conventional optical fibers made from silica and its doped materials, where light-guiding is achieved through total internal reflection, MOFs and their group of hollow-core MOFs (HC-MOFs) represent a separate class of photonic bandgap fibers, for which the guidance is accomplished by coherent Bragg scattering [8,33] that forms well-defined permitted and prohibited regions for photon propagation within the central core of the fiber [1,15]. This results in the appearance of transmission peaks and dips in the spectra of HC-MOFs, demonstrating that only specific wavelength bands are confined into the central core and allowed to propagate [34,35]. The transmission spectra of HC-MOFs can feature either single or multiple peaks depending on the specific MOF structure and material composition [36]. The light-guiding mechanism in such fibers is described via Fabry-Perot resonances. In accordance with this model, the maximal decoupling of the core and cladding modes that correspond to the maxima in the fiber transmission occurs at:

(1) $$\lambda_{j}=\;\frac{4n_{1}}{2j+1}{\left(\frac{n_{2}^{2}}{n_{1}^{2}}-1\right)}^{1/2}.$$

where j is an integer describing mode order (*j* = 1, 2, 3,…), *n*_1_ is RI of an analyte filling the capillaries, *n*_2_ is RI of the fiber glass, and d indicates the wall thickness for the first capillary layer.

Solid-core MOFs exploiting the principle of total internal reflection for light guidance in a high refractive index core, represent the major group of these fibers, however, they can also guide light in a low refractive index core based on the photonic bandgap guidance [37].

**Figure 4:**
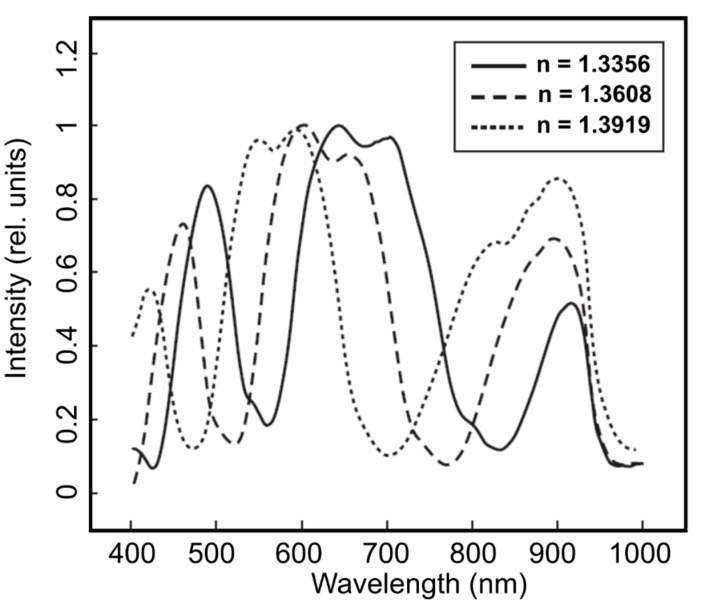
Transmission spectra of MOF samples filled with glucose solutions with different refractive indices. The change of glucose concentration from 1% to 40% induces the refractive index (n) variation in the interval 1.3356–1.3919 (at 20 °C). Reproduced with permission from [34].

In addition to all the other advantages and features of OFSs, MOFs with their capability to guide light in the HC region and the strong interaction with an injected medium open new perspectives for the applications of OFSs, which are not possible with all-solid fibers [32,38,39,40]. The liquid filling of hollow capillaries of MOFs, enables in-fiber microfluidic optical sensing, measurements of the fluid’s refractive index [31,34,35], temperature [41], and fluorescence signals [42]. The high sensitivity to the change of solution refractive index enables the precise measurement of concentration in a very little analyte volume needed for the test (Figure 4).

The sensing with the solid-core MOFs is realized based on the strong interaction of the evanescent field of the propagating light mode with the air channels surrounding the central core. Among the other types of solid-core fibers, suspended-core MOFs are the most promising structures for efficient biological sensor devices, due to the high power fraction of the evanescent-field [37,43].

**Figure 5:**
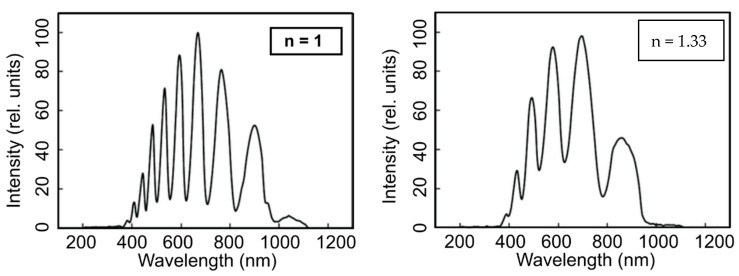
Transmission spectra of an empty (unfilled, n_air_ = 1) MOF sample (**a**) and one filled with water (n_water_ = 1.33) (**b**). Reproduced with permission from [35].

An additional advantage emanating from the structure of the MOFs, is their potential for the investigation of different liquids, through the selective or complete filling of air capillaries. Due to the liquid filling, the shift to shorter wavelengths or the blue shift will appear for the transmission bands compared to the unfilled fiber (Figure 5) [44]. This can be expressed as follows [32]:

(2) $$\lambda_{fill}=\lambda \sqrt[{}]{\frac{n_{glass}^{2}-\;n_{fill}^{2}}{n_{glass}^{2}-1}}$$

where *λ* is the wavelength of the unfilled MOF, $n_{glass}^{}$ is the RI of the fiber glass, and $n_{fill}^{}$ is the RI of the filling.

## 3. Selective Functionalization of Air Channels of Microstructured Optical Fibers

The selective filling of core and cladding air channels of hollow-core MOFs allows one to create a hybrid MOF-based structure in which the cladding is nearly all air and the central light-guiding hollow-core can be made from almost any solution-based material that is of particular importance for spectroscopy and sensing applications in aqueous solutions. One can also change the light confinement mechanism from photonic bandgap to total internal reflection guidance. Moreover, the control of the number of guided modes can be achieved with a proper choice of the inserted liquids. This technique can also be utilized to study the birefringent properties of MOFs [45]. An example of single-mode guidance in hollow-core MOF was demonstrated by Matos et al. for a particular choice of filled liquids [46]. 

Schwuchow et al. reported a functionalization technique, which allows for the selective deposition of different plasmonic nanoparticles in different air capillaries of the suspended-core MOF [47]. This method can potentially enhance the functionality of such fibers through the simultaneous detection of two different biomolecules, that are sucked individually into functionalized air channels.

**Figure 6:**
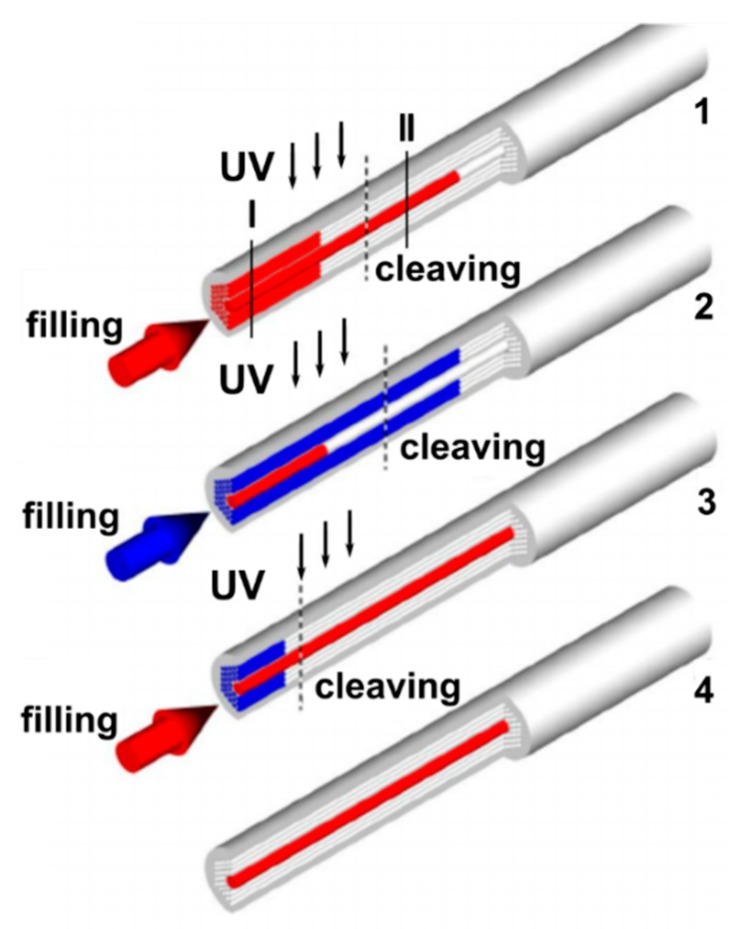
Schematic showing the selective filling of the central hollow-core and air-channels of the cladding of a MOF sample. Reproduced with permission from [48]. **1**—The optical ultraviolet (UV)-cured adhesive is injected into the air channels of the MOF (core and cladding capillaries) with a syringe (or by the capillary action). The liquid fills the central hollow-core much faster than the smaller cladding holes; consequently, after some point, only the central hollow-core becomes filled with the optical polymer. A UV lamp is applied to cure the optical adhesive inside the MOF and then the fiber is cut at the position called “cleaving” in such a way that only the central hole is filled by the cured polymer. **2**—The air cladding holes of the modified MOF structure are selectively filled with the optical adhesive while the central hollow-core remains blocked by the already cured polymer. The optical adhesive in the cladding holes is cured by the radiation of the UV lamp and the fiber is cut at the position called “cleaving”. At the end of the second step, all cladding holes were filled with cured polymer but the central hole was open. **3**—The central hole is selectively filled with the liquid while the cladding holes are plugged. **4**—After cleaving, the hybrid MOF with the central hole filled with the desired functional material is obtained while the fiber cladding consists of an array of air holes.

Among others, the most popular methods used for the selective filling of the MOF’s air capillaries, are the collapse of the cladding holes by a fusion splicer, as well as infiltration by temperature or UV-cured optical polymers inside the air channels of the fibers (Figure 6) [48]. 

## 4. Biosensors for the Selective Detection of Specific Molecules and DNA Based on Functionalized Microstructured Optical Fibers

**Figure 7:**
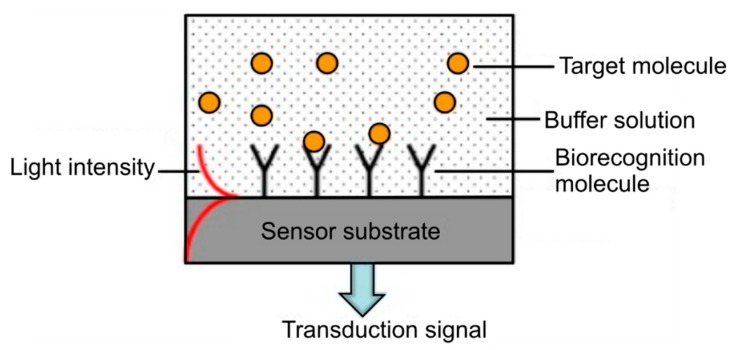
Illustration of the concept of a label-free optical biosensor. Reproduced with permission from [53].

MOF biosensitivity is achieved through the selective adsorption of target molecules on to fiber surfaces, that have been functionalized with antibodies in order to bind to specific antigens which are investigated (Figure 7) [14,49,50,51,52,53]. Specific adsorption-based sensors are used for human disease diagnosis and prevention with a sensitivity that meets clinical needs [23,54]. Further improvement of the performance of MOF-based sensors, can be accomplished by the variation of the geometry and material of the MOFs through the adjustment of the fiber parameters to a specific application. A smaller core size, increases the overlap of the propagating light mode with the analyte under test, effectively increasing sensitivity and decreasing the required sample volume [52]. Proper selection of the fiber material can also increase the numerical aperture of the fiber and consequently the proportion of the fluorescent signal that can be recaptured [52].

**Figure 8:**
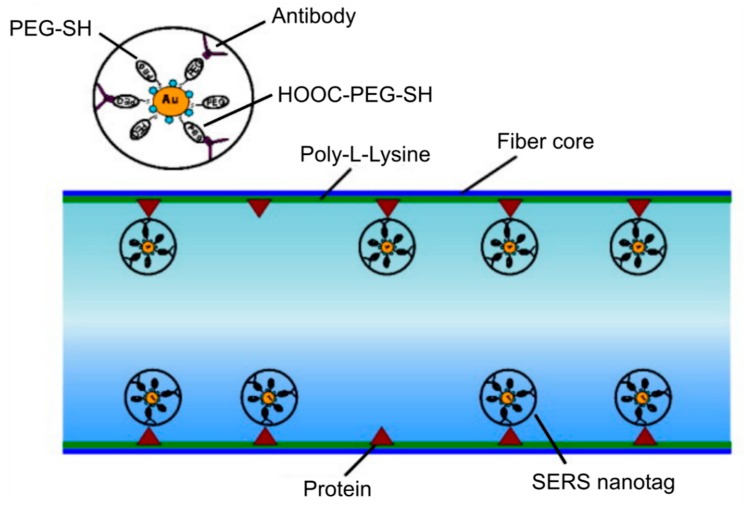
Sketch of the functionalized fiber surface showing the binding of anti-epidermal growth factor receptors (anti-EGFR) antibody conjugated surface-enhanced Raman scattering (SERS) nanotag to the cancer protein (positive human epithelial carcinoma cells A431) immobilized on the inner wall of the core of MOF. Reproduced with permission from [14].

Dinish U.S. et al. have reported a novel MOF-based surface-enhanced Raman scattering (SERS) sensor for the detection of cancer proteins in a very low sample volume [14] that can potentially be used for multiplex detection of biomarkers that are immobilized inside the hollow-core MOFs [54]. Ultrasensitive measurement of protein was achieved using anti-epidermal growth factor receptors (anti-EGFR) antibody conjugated SERS nanotag (Figure 8) and the simultaneous detection of hepatocellular carcinoma biomarkers-alpha fetoprotein and alpha-1-antitrypsin secreted in the supernatant from the Hep3b cancer cell line was demonstrated [54]. It has been shown that the proposed detection method is sensitive to a low amount of proteins at ~100 pg in a sample volume of ~10 nL. 

Biological sensors based on modified MOFs have also found application in selective DNA detection. The functionalization of the fiber surface leads to the binding of biological species to the glass surface that is then proved through the measurement of the fluorescence signal created by the labeled sample [37].

**Figure 9:**
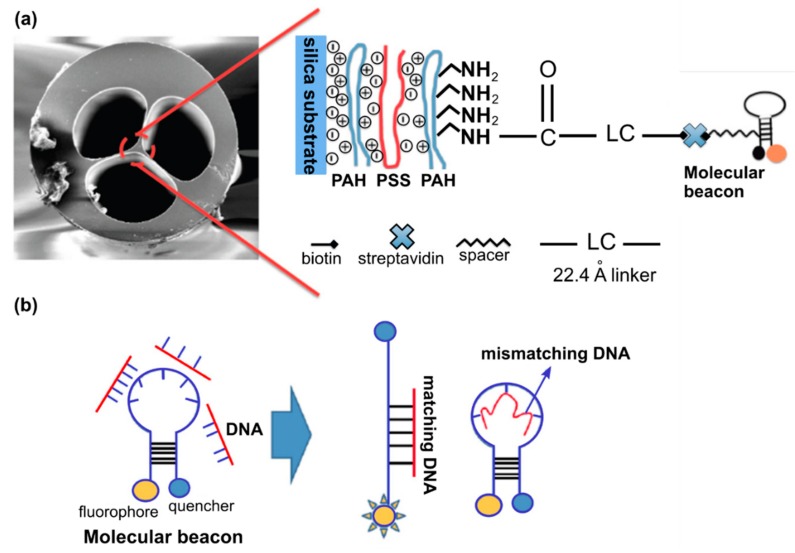
(**a**) SEM image of MOF end face and schematic representation of the modification process of the MOF surface. (**b**) Molecular beacons conformational change upon hybridizing with cDNA while remaining in closed form upon hybridizing with nDNA. Adapted with permission from [57]. © The Optical Society.

Several groups reported the feasibility of MOF-based biosensor for DNA detection [55,56]. Ngyuen L.V. et al. proposed that functionalized MOFs can act as a highly specific DNA sensor and experimentally demonstrated the detection of DNA in nanoliter-scale sample volumes [57]. The modification of the fiber surface consisted of a combination of the fuzzy nano assembly technique named also layer-by-layer assembly method [58] and the biotin-streptavidin binding mechanism (Figure 9). The authors showed that the created sensor allows for the detection of DNA solutions at a concentration of 4 µM with the potential for further improvement.

**Figure 10:**
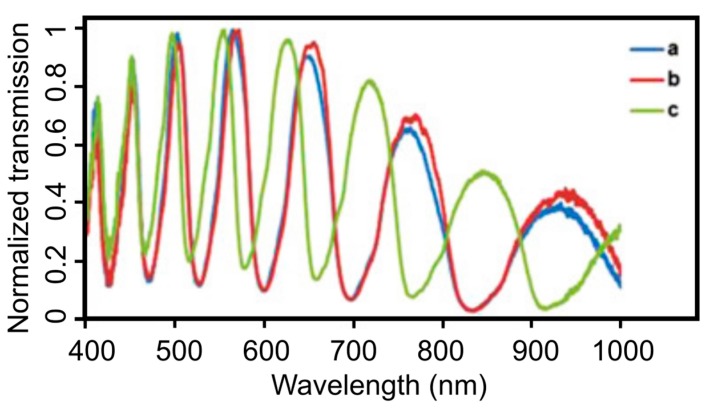
Transmission spectra measurements of functionalized MOFs. (**a**) Unmodified MOF. (**b**) MOF sample treated with a concentrated H_2_SO_4_ solution and (**c**) in a mixture of H_2_SO_4_ and H_2_O_2_ (50:50 volume ratio). Reproduced with permission from [60].

Among the other modification procedures, the silanization of the fiber inner walls has been shown to be the most stable approach for the binding of biomolecules to silica surfaces [37,56,59]. Pidenko S.A. et al. estimated the amount of silanol groups on the inner surfaces of HC-MOFs after its hydroxylation with a mixture of concentrated sulfuric acid (H_2_SO_4_) and hydrogen peroxide (H_2_O_2_) by the transmission spectra measurements of modified MOF samples and the analysis of the spectral shift of the maxima of the local transmission band in the visible spectral region (Figure 10) [60]. The possibility for the creation of bio sensitive structures based on functionalized MOFs was shown for the covalent binding of horseradish peroxidase to the obtained silanol-modified fiber surface.

Coscelli E. et al. reported the functionalization technique of the inner surfaces of the hollow channels of the suspended-core MOFs that allows for the selective detection of DNA through the hybridization of immobilized peptide nucleic acid probes [37].

Kostecki R et al. realized the novel one-step polymer functionalization method for the creation of a MOF-based sensor [61]. This process eliminates the need for the functionalization of the fiber surface with the functional groups for sensor molecule attachment but alternatively, it combines the polymer, silica, and sensor molecule elements for a distributed sensor to allow for the detection of an analyte along the length of the whole fiber. This approach was successfully tested for the detection of Al cations in solution by doping the poly(methyl methacrylate) film with 8-hydroxyquinoline sensing molecules [61]. Another prospective application of functionalized MOFs is the specific detection of target molecules or blood components in point-of-care devices [62]. 

## 5. Microstructured Optical Fibers Functionalized with Plasmonic Nanoparticles and MOF-Based Optofluidic Platforms

The air capillaries, which run along the whole MOF structure, act as small sample reservoirs and allow for liquid sensing in the nano- and microliter scale. The proximity of the hollow channels surrounding the central solid-core of the suspended-core MOFs enables a strong overlap of the light guided mode and any material filled inside. The major advantage of using hollow-core MOFs rather than other techniques based on geometry-modified optical fibers, cuvettes, and bulk optics, lies in combining the long interaction lengths with strong overlapping between the light mode, that penetrates deeply into the air capillaries via its evanescent field, and the injected analyte [16]. More practical approaches of using the accessibility of the air capillaries for the functionalization of the MOF surface were shown by Sukhishvili S. et al. on the example of solid and hollow-core MOFs [50,63] (Figure 3a,c). Effectively, the proposed methods combine both the advantages of microfluidics and fiber optics in a single MOF sample, allowing for light guidance with simultaneous liquid flow inside the hollow capillaries. The authors showed the fine accumulative SERS signal from the full-length Ag-nanoparticle functionalized MOFs, as well as the potential for fine control of the density of deposited Ag nanoparticles and studied the SERS gain and light attenuation of the Raman intensity with MOF samples of different length [50]. Csaki A. et al. demonstrated the technique for the preparation of finely tuned plasmonic layers of Ag-nanoparticles inside the capillaries of suspended-core MOFs [64] (Figure 3b). They employed a combination of microfluidics and self-assembled monolayer method, leading to a uniform deposition of silver nanoparticles, enabling precise control of the spectral characteristics of the functionalized MOF sample by the selection of Ag-nanoparticles with known plasmonic characteristics [64]. The authors reported the enhancement of the Raman signal for a MOF sample functionalized with Ag-nanoparticles compared to unmodified fibers, by investigating crystal violet as a model substance [65]. The possibility of integrating colloidal bimetallic nanoparticles with predefined parameters into HC-MOFs (Figure 3c) was demonstrated by Ponce S. et al. [66]. With the proposed method, PtNi clusters became strongly attached to the inner surface of the hollow-core and could be used as active catalysts for the hydrogenation of an azobenzene dye, opening new perspectives for in situ catalyst monitoring. They have also showcased that the optical transmission dependents on the size of nanoparticles, i.e., larger nanoparticles result in more optical signal loss. 

**Figure 11:**
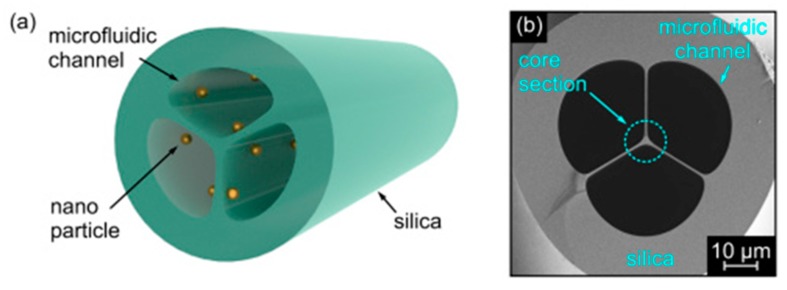
(**a**) Schematic showing the suspended-core MOF functionalized with plasmonic nanoparticles. (**b**) SEM image of the central microstructured section of the investigated MOF. Grey and black areas correspond to silica glass and air, respectively. Adapted with permission from [67]. © The Optical Society.

Another example of the MOF-based optofluidic platform has shown that suspended-core MOFs functionalized with gold nanoparticles, can be utilized for real-time analyte monitoring through efficient RI sensing (Figure 11) [67,68]. 

The concept of low-cost microfluidic-compatible sensing platform for fast detection of small RI variations was realized with exposed-core optical fibers functionalized with plasmonic gold nanoparticles [69]. MOF-based biosensors for the reversible and low volume scale measurement of metal ions were proposed and experimentally realized by Monro T. et al. [70,71,72]. The new sensor structure was highly selective to ion-binding, while also allowing sampling of small volumes. The possibility for the sensor to be used for the series of experiments without the need to change it, which is of particular importance in biophotonics, was also shown. They demonstrated the relevance of the proposed sensor type in real-time or in situ detection of zinc, aluminum, and lithium ions; however, the approach is versatile, with the potential usage for the detection of other ions as well. The recent development and applications of MOFs in the microfluidic field was reviewed by Shao L. et al. [73], Tian F. et al. [74], Eggleton B. J. et al. [75], and Pissadakis S. and Selleri S. [76].

## 6. Hybrid Microstructured Optical Fibers

**Figure 12:**
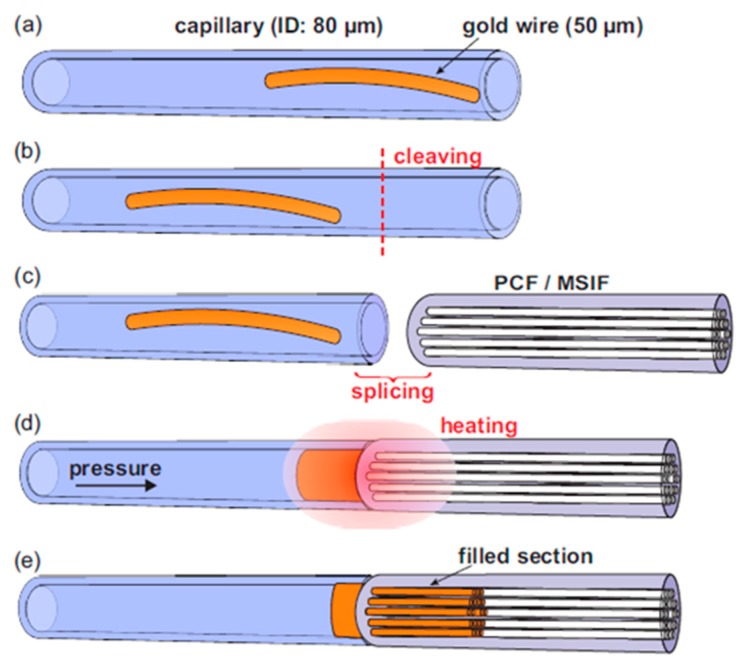
The schematic of the technique of pressure-assisted melt filling of the air channels of MOF samples. (**a**) The gold wire is inserted into the host capillary. (**b**) The fiber end face is cleaved. (**c**) The peace of the MOF sample is spliced to the host capillary. (**d**) The whole structure is placed into the furnace with the simultaneous connection of the host capillary to a pressure chamber. (**e**) The resulting structure of the hybrid optical fiber. Adapted with permission from [79]. © The Optical Society.

The further development of MOF-based sensors gave rise to a new research direction for the tuning of optical properties. Various approaches have been proposed and realized for MOF modification [77]; different solid [15,16,78,79,80,81] and liquid materials [82] were injected into the fiber hollow regions. Among others, one can highlight such well-described approaches for the injection of host materials such as pressure-assisted melt filling (Figure 12) [79], chemical vapor deposition [83], and direct fiber drawing [77]. 

Schmidt M. et al. reported different hybrid MOFs realized though the selective or complete filling of the fiber air channels by the metal wires or molten glasses. The authors investigated the material and optical properties of solid-core MOF with integrated micron-sized Ge wire [84]. The series of clear dips in the transmission spectrum of functionalized MOF samples was attributed to the anti-crossing between the propagating optical mode in the glass core and the resonances on the high index Ge wire and this allowed the construction of different kinds of in-fiber detectors and sensors. The possibility for supercontinuum generation in the modified fibers was demonstrated for a silver metaphosphate/silica step-index fiber, as well as an arsenic trisulphide waveguide embedded in silica produced by pressure-assisted melt filling [85,86]. Markos C. et al. realized the thermo-tunable hybrid MOF in which air-channels were functionalized with arsenic trisulfide glass nanolayers [87].

The generation of high power CW-lasing and the amplification of nanosecond pulses were realized in the MOF samples whose glass materials were doped with Thulium (Tm) [88,89] and Erbium (Yb) [90]. 

## 7. Microstructured Optical Fibers Coated with a Layer-By-Layer Assembly of Inversely Charged Polyelectrolyte Layers

Recently, the technique of polyelectrolyte LBL deposition, originally applied for the preparation of nanofilms [13,58] and later used for the formation of microcapsules [91,92,93,94], as well as the functionalization of planar surfaces [95], have been adapted for the surface modification of optical fibers [96]. These can either be buffer layers [15,16] with a controlled value of surface potential for better particle adsorption or the sensitive layers by themselves [97]. A nanoscale thickness accuracy is possible by varying a set of parameters such as, among others polyelectrolyte concentration, adsorption time, ionic strength, solvent composition, and temperature [58].

**Figure 13:**
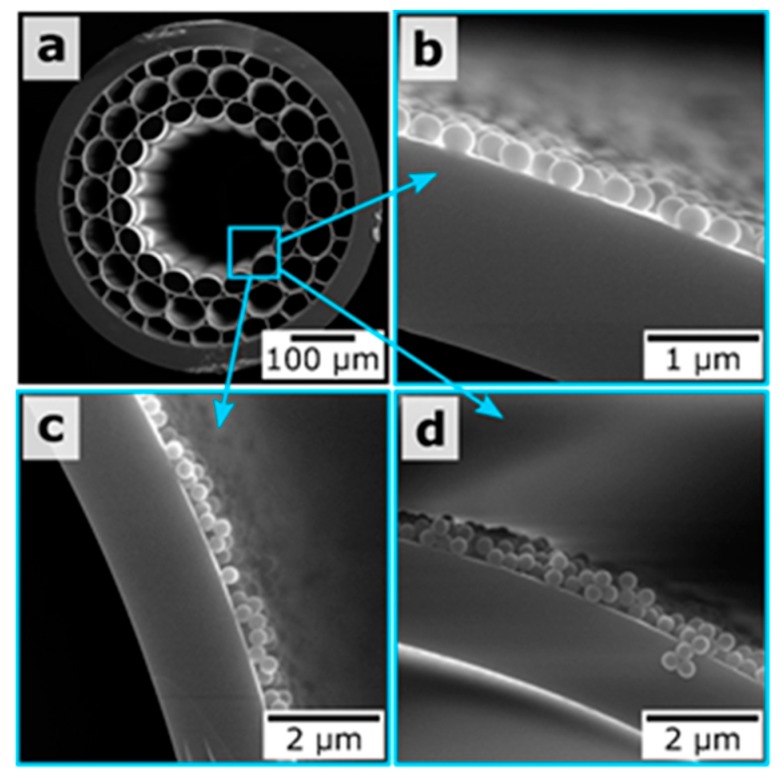
(**a**) SEM image of the MOF end face. (**b**–**d**) Magnified SEM images of the hollow-core regions for the MOF samples coated with one, two, and three layers of 300 nm silica particles, respectively. Reproduced with permission from [16].

A technique that allows magnetic resonance imaging of hollow-core MOF samples was demonstrated for the case of LBL assembly of oppositely charged polyelectrolytes and magnetite nanoparticles on the inner surface of hollow-core, opening new prospects for fiber-based endoscopic devices with magnetic resonance imaging that can potentially lead to minimally invasive medical diagnostics and surgical procedures in vivo [15]. Based on a similar approach of host materials deposition inside MOF samples, we reported a novel type of functionalized MOF sample whose capillaries were coated with silica submicron particles (SiO_2_) with different diameters (300, 420, and 900 nm) and layers of poly(diallyldimethylammonium chloride). We also recently demonstrated the possibility of multilayer deposition on the example of silica particles at a diameter of 300 nm (Figure 13) [16]. This modification technique of MOFs allows one to reach novel sensing capabilities, which benefit from an increased effective sensing area and the provision of a convenient scaffold for the binding of specific molecules [14].

## 8. Microstructured Optical Fibers Functionalized with Fluorescent Nanoparticles

**Figure 14:**
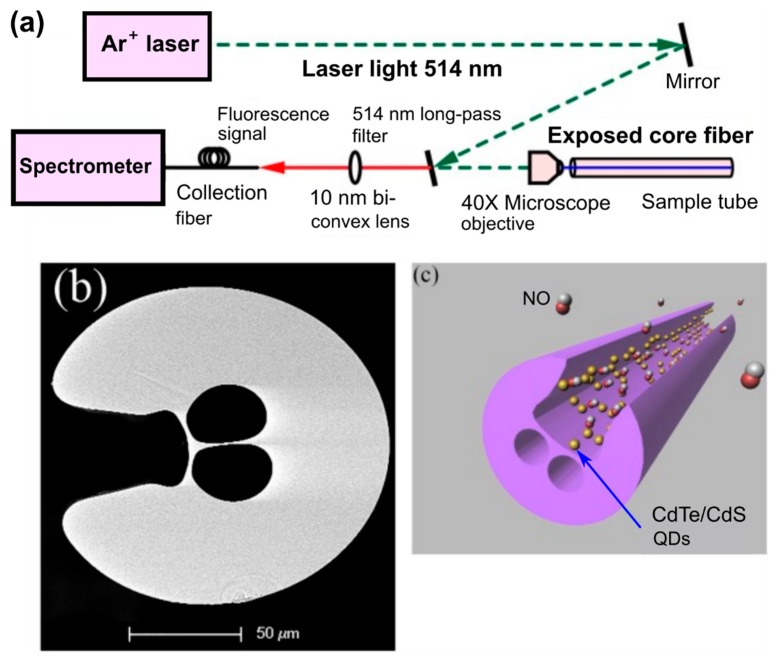
(**a**) Schematic of the experimental setup used for the sensing on the nitric oxide. (**b**) SEM image of the cross-section of the exposed-core MOF sample used in the experiments. (**c**) Illustration of the proposed sensor structure based on functionalized MOF sample. Reproduced with permission from [102].

The effect of the hollow-core MOFs on to the emission wavelength and the amplitude of the fluorescent nanoparticles leads to the study of the potential applications of integration of the quantum dots into the hollow-core region [98,99]. Bozolan A. et al. realized the temperature sensor based on MOF modified by the CdSe/ZnS nanocrystals through the measurements of their luminescence spectrum [41]. Larrion B. et al. monitored the optical absorption, the emission intensity, and the emission peak wavelength in the range from −400 to +700 °C of the MOFs with integrated CdSe quantum dots [100]. Mak S.W.J. et al. demonstrated the great potential for the application of HC-MOFs for optical sensing with low analyte volume. They observed the clear vibrational modes of the CdTe core, CdS_0.7_Te_0.3_ interface, and carboxylate-metal complexes in dilute aqueous CdTe quantum dot solutions using the MOF-based structure [101]. Monro et al. reported a novel approach for the detection of nitric oxide using an exposed-core microstructured optical fiber coated with CdTe/CdS core/shell quantum dots [102]. The detection of nitric oxide, which plays an important role in biological systems, but is restricted because of its relatively low concentration and short half-life time, opens up the possibility for monitoring its production within biological systems (Figure 14). The authors showed that MOF samples functionalized with CdTe/CdS quantum dots could respond rapidly to nitric oxide with picomolar sensitivity.

## 9. Thermography

Monitoring of local temperature in individual organs of the human and animal body with high precision is of great importance in physiology for understanding the pathogenesis of many diseases and accompanying treatment by measuring the temperature in tumors or tissues, for example, during laser ablation or other photothermal treatments. Obviously, fiber-optic systems are particularly well suited to solve these problems [103,104,105,106,107]. For example, measuring the temperature in the brain requires a spatial resolution of a sub-millimeter and a temperature resolution of less than 0.5 °C. Fiber-optic temperature sensors can be used to measure in vivo temperature in the brain of animals during their free behavior and movement [103]. The ability of such sensors to measure temperature in the range from the room and normal body temperature (36–37 °C) to ablative (100 °C) with a temperature resolution of about 0.1 °C is desirable when creating new endoscopic systems for obtaining thermal images [105,106]. The most common fiber-optic methods for measuring temperature are described in numerous literature (see, for example, [103]), many of them are not ideal for biosensing due to low spatial resolution or increased sensitivity to the refractive index of the environment.

For the first time, the presence of a bandgap above 3 μm in a silica-based air-core photonic crystal fiber (PCF) with a bandgap peak of 3.14 μm and a typical attenuation of ~2.6 dB/m was reported in [104]. Such microstructured hollow-core fibers were considered as an alternative to optical fibers based on fluoride, tellurite, or chalcogenide glass. Silica-based PCFs are especially important for use in biological sensors of the mid-IR range [104]. The most critical advantage of hollow-core fibers (HCFs), i.e., photonic bandgap and antiresonance fibers, is that the propagating light is confined within the hollow-core, so it is not very dependent on the optical properties of the material. Such fibers have already been used for thermal imaging. Not only pure silica mid-IR HCFs have been fabricated [107], but also HCFs coated with a metal film or dielectric material on the inner surface of the capillary tube (see [107]).

**Figure 15:**
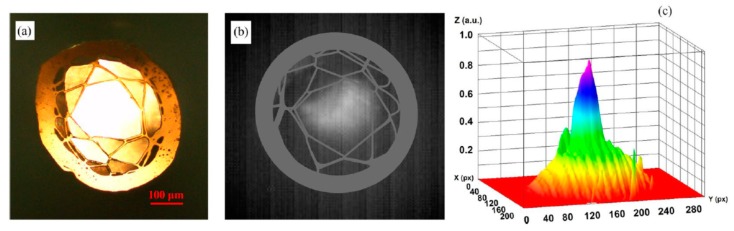
(**a**) Hollow-core fiber cross-section under an optical microscope operating in the visible, (**b**) mid-IR mode image, and (**c**) intensity profile. Reproduced with permission from [107].

A recent paper [107] demonstrated robust HCF drawing technology from a 3D printed fiber preform, which is capable of guiding light in the mid-IR range. For printing a hollow-core preform, a transparent filament of glycol polyethylene terephthalate (PETG) was processed by using the fused deposition method (FDM). Despite the fact that PETG has a material absorption exceeding 10 dB/mm in the spectral range of 3.5–5 μm, the light in the HCF is guided by the antiresonance confinement, resulting in propagation loss two orders of magnitude smaller than the PETG absorption loss. The final outer diameter of the PETG fiber was 466 μm, and the diameter of the hollow-core was 225 μm. Thermal imaging on the fiber facet, performed in the wavelength range of 3.5–5 μm, clearly indicates air guidance in the fiber hollow-core. In order to evaluate the waveguide properties of the HCF in the mid-IR, the radiation of a broadband lamp (Thorlabs SLS202, 450–5500 nm) was launched into a 12 cm long section of fiber using a bare-fiber adapter. The modal image at the output of the fiber in the wavelength range λ = 3.5–5 μm was obtained using a thermal infrared camera (Onca-MWIR-Insb) using a ZnSe lens with a focal length of 18 mm. Figure 15 shows a thermal image and its intensity profile at the output of HCF. In Figure 15b it is clearly seen that infrared light is guided in the air-core, which is caused by antiresonance reflection in the first layer of polymer strands (Figure 15b,c). The HCF mid-IR guiding properties was also proved by the near-field imaging of the transmitted light under different degrees of fiber bending that varied from 0 to 45° with the radius of curvature of ~76 mm.

The measured propagation loss of 30 dB/m was about two orders of magnitude smaller than the loss of polymer material. Propagation loss can be improved by optimization of printing and fiber drawing processing technology.

For future development of HCF systems suitable for biological thermography, the technology described in References [105,106] can be useful. In these papers, the high-resolution hollow-core coherent mid-IR fiber bundles for endoscopic infrared imaging in the 8–10 μm spectral range were designed. Authors employed the hollow glass waveguide technology with Ag/AgI thin film coatings, and claimed that multilayer dielectric coated hollow waveguides can have much lower loss than single layer coated waveguides, and correspondingly the bore size of the tubing in the bundle will be smaller and lead to better spatial resolution.

To achieve high accuracy in temperature measurement, of 0.1 °C, upconversion visible luminescent thermometry can be used, where rare-earth ions such as erbium or thulium are doped within a host medium such as HCF material [103]. The most advantage of detection of the upconversion luminescent in living systems is that there is no autofluorescence as the excitation is in the near infrared (NIR) and luminescent is in the visible.

## 10. Summary of the Reviewed Functionalized Techniques

An overview of the existing functionalization techniques and applied materials [108], their comparison, and the application areas can be found in References [32,38,42,52,103,109]. Table 1 summarizes the reviewed techniques applied for MOFs functionalization, their potential applications, and describes the used MOF types.

## 11. Conclusions

In this work, we overviewed the existing types of MOF modification techniques, with a focus on the ones that improve the performance of MOF-based sensors and open new prospects for their usage. We described the methods of integrating the host materials inside the fibers on the example of plasmonic nanoparticles and nanowires. The application of the proposed structures for the detection of specific molecules and for the monitoring of the refractive index variation of the analyte in the test has been discussed. The LBL assembly approach, which was adapted for the functionalization of MOFs and the creation of sensitive and adhesion layers for target particle adsorption, has been considered as well. Different methods applied for the modification of optical fibers are summarized in Table 1, which highlights the novel applications of MOFs that are beyond the manipulation of transmitted light but reveal new kinds of functionalities that could be achieved with these structures.

## Figures and Tables

**Table 1:** Summary of the reviewed MOF functionalization technique and their potential applications.

MOF Type	Functionalization	Application	Ref.
Suspended-core	Selective deposition of different plasmonic nanoparticles into different hollow channels surrounding the central solid-core	Simultaneous detection of two different biomolecules	[47]
Hollow-core	Selective filling of core and cladding air channels	Control of the number of guided modes; single-mode guidance	[45,46]
Hollow-core and suspended-core	Functionalization of fiber surfaces with antibodies specific binding to antigens under test	Specific adsorption-based sensors for human disease diagnosis and prevention; selective adsorption of the target molecules on to fiber surfaces; MOF-based SERS sensor	[14,54]
Suspended-core	Combination of the fuzzy nano assembly technique and the biotin-streptavidin binding mechanism; hybridization of immobilized peptide nucleic acid probes	Biosensor for selective DNA detection based on suspended-core MOF	[37,55,56,57]
Hollow-core	Silanization of the fiber inner walls	Creation of biosensitive structure on the example of the covalent binding of horseradish peroxidase to the obtained silanol-modified fiber surface	[60]
Solid-core and hollow-core	Finely tuned plasmonic layers of Ag-nanoparticles inside the air capillaries of MOFs; a combination of microfluidics and self-assembled monolayer method, leading to a uniform deposition of silver nanoparticles	Fine accumulative SERS signal from the full-length Ag-nanoparticle functionalized MOFs and fine control of the density of deposited Ag nanoparticles	[50,63,64,65]
Hollow-core	Integration of colloidal bimetallic nanoparticles with predefined parameters into HC-MOFs	In situ catalyst monitoring	[66]
Suspended-core	Gold nanoparticles-functionalized suspended-core MOF	Efficient RI sensing featuring the real-time analyte monitoring	[67,68]
Suspended-core and exposed-core	Functionalization of hollow channels with a monoazacrown bearing spiropyran; attachment of the fluorophore to a polyelectrolyte-coated fiber’s surface	Biosensors for the reversible and low volume scale measurement of metal ions; real-time detection of zinc, aluminum, and lithium ions	[70,71,72]
Solid-core	Integrated micron-sized Ge wire inside the air channel of modified step-index MOF	In-fiber detector and sensor	[84]
Solid-core and hollow-core	Silver metaphosphate/silica step-index fiber and an arsenic trisulphide waveguide embedded in silica produced by pressure-assisted melt filling	Supercontinuum generation	[85,86]
Hollow-core	Doping glass materials with Thulium (Tm) and Erbium (Yb)	Generation of high power CW-lasing and the amplification of nanosecond pulses	[88,89,90]
Hollow-core	LbL assembly of inversely charged polyelectrolytes and magnetite or silica particles at different diameters	Magnetic resonance imaging of hollow-core MOF; increased effective sensing area and the provision of a convenient scaffold for the binding of specific molecules	[15,16]
Suspended-core and hollow-core	Coating with quantum dots on the inner surfaces of hollow channels	Temperature sensor based on modified MOF sample by the CdSe/ZnS nanocrystals; detection of nitric oxide by using an exposed-core MOF coated with CdTe/CdS core/shell quantum dots	[41,101,102]
